# A validation study for a bat-inspired sonar sensing simulator

**DOI:** 10.1371/journal.pone.0280631

**Published:** 2023-01-20

**Authors:** Hongxiao Zhu, Anupam Kumar Gupta, Xiaowei Wu, Michael Goldsworthy, Ruihao Wang, Mohitha Mikkilineni, Rolf Müller

**Affiliations:** 1 Department of Statistics, Virginia Tech, Blacksburg, Virginia, United States of America; 2 Department of Engineering Maths and Bristol Robotics Laboratory, University of Bristol, Bristol, United Kingdom; 3 Department of Computer Science, Virginia Tech, Blacksburg, Virginia, United States of America; 4 Department of Mechanical Engineering, Virginia Tech, Blacksburg, Virginia, United States of America; 5 Department of Electrical and Computer Engineering, Virginia Tech, Blacksburg, Virginia, United States of America; Doshisha University, JAPAN

## Abstract

Many species of bats rely on echoes to forage and navigate in densely vegetated environments. Foliage echoes in some cases can help bats gather information about the environment, whereas in others may generate clutter that can mask prey echoes during foraging. It is therefore important to study foliage echoes and their role in bat’s sensory ecology. In our prior work, a foliage echo simulator has been developed; simulated echoes has been compared with field recordings using a biomimetic sonar head. In this work, we improve the existing simulator by allowing more flexible experimental setups and enabling a closer match with the experiments. Specifically, we add additional features into the simulator including separate directivity patterns for emitter and receiver, the ability to place emitter and receiver at distinct locations, and multiple options to orient the foliage to mimic natural conditions like strong wind. To study how accurately the simulator can replicate the real echo-generating process, we compare simulated echoes with experimental echoes measured by ensonifying a single leaf across four different species of trees. We further extend the prior work on estimating foliage parameters to estimating a map of the environment.

## Introduction

Many bat species rely on echolocation—they emit short ultrasonic pulses and listen for the returning echoes to support navigation and prey hunting [1]. The dominant frequency in bat biosonar pulses can reach up to 212 kHz [2] with thresholds for object detection as low as 0.05 mm [3]—smaller than the thickness of human hair. The extremely capable sonar sensing system coupled with low energy requirements makes bats an excellent biological model for the study of smart sonar systems.

As many bat species inhabit vegetated environments such as forests, foliage echoes play an important role in bats’ sensing experience. Natural foliage is indeed challenging for sonar sensing—it consists of a multitude of sound-reflecting surfaces which can produce interference that masks a target echo [4, 5]. Previous studies suggest that foliage echoes are highly stochastic [6] and that bats rely on statistical features of the echoes for object/target recognition/detection [7–11]. These studies, however, were based on experiments with animals or biomimetic sonar, which limits the amount of the available data. As a result, the analysis have been focused on simple tasks such as plant classification [8, 12], texture recognition [7], landmark identification [11, 13, 14], and passageway finding [9, 10]. An alternative approach is to use simulations to recreate a much wider range of sensing scenarios. A simulation approach can produce foliage echoes based on full knowledge of the (simulated) foliage geometry, which makes it possible to study more complicated sensing scenarios that are beyond the scope of physical experiments. For example, imagine training an Unmanned Aerial Vehicle to fly through a forest based on biomimetic sonar. This would require acquiring large numbers of foliage echoes along with the respective location data. Simulation offers a convenient method for such study that is not only cost-effective (compared with experiments) but also easily repeatable.

Several computer simulation models have been developed to replicate the physical processes that underlie the foliage echo creation. For example, authors in [8] presented a computational foliage model that treats leaves as point reflectors. This work considered the spatial distribution of reflectors but did not take into account factors such as leaf shape, size, orientation, and occlusion. A more recent work by [15] proposed a computational model for foliage echoes that accounts for the density, size, and orientation of leaves that were left out by [8]. In both [8, 15], foliage echoes are simulated as superposition of echoes returned from reflectors. A follow-up work by [16] investigated several simplifications made in [15]. Specifically, this work studied the effect of approximating leaf shape with a disk and assessed whether the uniform leaf distribution assumed in [15] needs to be relaxed to include inhomogeneous foliage patterns simulated from Lindenmayer-system model [17]. More extensive studies on the simulation of random trees have been done by [18]. A more recent work of [19] introduced a unified simulation framework that combines the simulation of natural forests with the foliage echo simulator of [15, 16]. This integrated simulation framework can be used for the training of robotic algorithms in biosonar-based Unmanned Aerial Vehicles. Based on simulated foliage echoes, various statistical estimation methods have been proposed to estimate relevant foliage parameters [16, 20, 21].

One key concern behind simulation-based approach is the fidelity in representing the real system. While it is reasonable to expect that any simulated echoes will exhibit certain deviations from the real foliage echoes due to necessary model simplifications, a valid model should retain key properties of the echo-generating process and produce data that are close enough to reality. A preliminary analysis of the effect of some of the model simplifications, such as treating leaves as hard discs and neglecting the shading between leaves, were carried out in [16]. However, there is still a lack of side-by-side comparison between simulated and experimental echoes under similar experimental setups. The goal of this paper is to fill this gap by presenting a validation study that compares outputs of the simulator with experimental data under similar experimental conditions.

We consider the simulator used by [15, 16, 19] as it is by far the most general foliage echo simulator established. To avoid introducing multiple uncontrollable factors, we collected experimental data under a simple setup—only single leaves from multiple species were ensonified and echoes were collected using a biomimetic sonar head. We made several modifications to the simulator to achieve a closer match with the biosonar experimental set-up as well as to enable the modeling of a wider range of sensing scenarios. We compare the simulation and experimental echoes both in time- and frequency-domain and use statistical summaries to characterize the differences. In addition to the comparison of echoes, we also demonstrate the application of our modified simulator on constructing a partial map of the surroundings based on foliage echoes.

The contributions of this work are as follows: 1) It demonstrates a close match between experimental and simulated echoes obtained from individual leaves, thereby supports the usage of the simulator to study more challenging scenarios hard to study experimentally. 2) It proposes an estimation approach to construct a partial map—the map of a specific region of the environment—through actively scanning the region. This is computationally more efficient than always mapping the whole environment, thus is more suitable for real-time navigation systems such as biosonar-based Unmanned Aerial Vehicles. 3) It proposes a modified simulator that not only achieves a close match with experimental echoes but also enables study of biosonar across a wider set of sensing scenarios such as object detection, obstacle avoidance, path planning, and target tracking.

## Materials and methods

### Foliage echo simulator

In this section, we briefly review the foliage echo simulation model previously established by [15, 16], and describe the modifications made to achieve a better match with the experimental data and to enable simulation of a wider set of experimental scenarios. The echo simulation model developed by [15, 16] has two components: (i) simulation of individual leaves and (ii) simulation of entire foliages. The leaves are modeled as round disks characterized by their radii and locations in a three-dimensional space. To generate foliage, leaf parameters such as radii, locations, and orientations can be generated from truncated uniform or normal distributions [15]. If more realistic foliage shape is desired, the spatial arrangement of the leaves (location and orientation) can also be modelled using L-systems [16, 19]. The leaf locations and orientations are later used to calculate the incidence angle for each leaf reflector—the angle between leaf normal and the axis connecting the emitter and the leaf center.

To reduce the computation time needed to produce the synthetic foliage echoes, the leaves that fall outside the sonar beam footprint (which is a function of the sonar beampattern and distance between the emitter and the foliage) are removed from the echo calculations beforehand. In addition, leaves that generate weak echoes (below -80dB) are also left out from computation. For this work, the sonar was assumed to be monostatic with identical directivity patterns for both emitter and receiver. The directivity patterns were approximated as the product of two Gaussians, one to model gain as a function of azimuth and the other for gain as a function of elevation angle.

The foliage echoes were simulated in the range of 60 to 80 kHz based on the emission recorded for greater horseshoe bats [22]. This choice, as well as other design of the simulator such as the sonar beampattern, are made to mimick bats’ biosonar from the biomimetic point of view. The basic principle of simulation is as follows: first, we calculate the frequency domain response for each leaf reflector at each frequency component in the range, and set the responses at other frequencies to be zero. This requires calculating the amplitude and phase delay at each leaf and at each frequency component. This calculation takes several parameters as inputs, including the sonar beampattern, the approximated leaf beampattern, and the distance between the sonar and the leaf center; second, the overall frequency domain components of the impulse response are calculated by summing over frequency components across all leaves; finally, we apply inverse Fourier transform to the frequency domain responses to obtain the time domain impulse response of the entire foliage. More details of the simulator can be found in [15] and the appendix of [20].

We improved upon the existing simulator [15, 16] in multiple ways. Prominent changes include: (1) The speaker and the microphone can now be placed at arbitrary locations, allowing for more accurate recreation of an experimental setup. (2) Multiple ways of orienting simulated leaves are introduced; for example, all leaves can now be oriented in the same direction on average to mimic conditions like strong wind blowing; alternatively, each leaf can have an orientation that is independent of others. (3) Separate spatial directivity patterns (beampatterns) are used for microphone and speaker in simulation to allow a better match with the experimental set-up. The directivity patterns for the microphone and speaker can be measured experimentally, or in the case of speaker, can also be analytically modeled as a circular baffled piston. If desired, the measured or modeled directivity patterns can be further simplified with a Gaussian approximation. (4) the output and input of the speaker and microphone are modulated by their respective frequency responses instead of the flat frequency response used previously. (5) The simulation now allows for generating directive patterns for a given beamwidth in degrees (beamwidth measured at -3dB from the peak of 0 dB) or an input frequency. (6) The location of the peak directivity can now be set at any arbitrary location within the frontal hemisphere, which allows for the study of problems that require sonar sweeps for detection/recognition or perusal of object. In summary, all above changes allow for a closer match between simulated and experimental setup, and allow for the study of a wider range of problems that were previously intractable. Notice that these improvements are rather generic and targeted at a large scope of situations. Some of them, such as (1) and (2), will not be used in the simpler setup of the current validation study.

### Experimental validation

**Fig 1:**
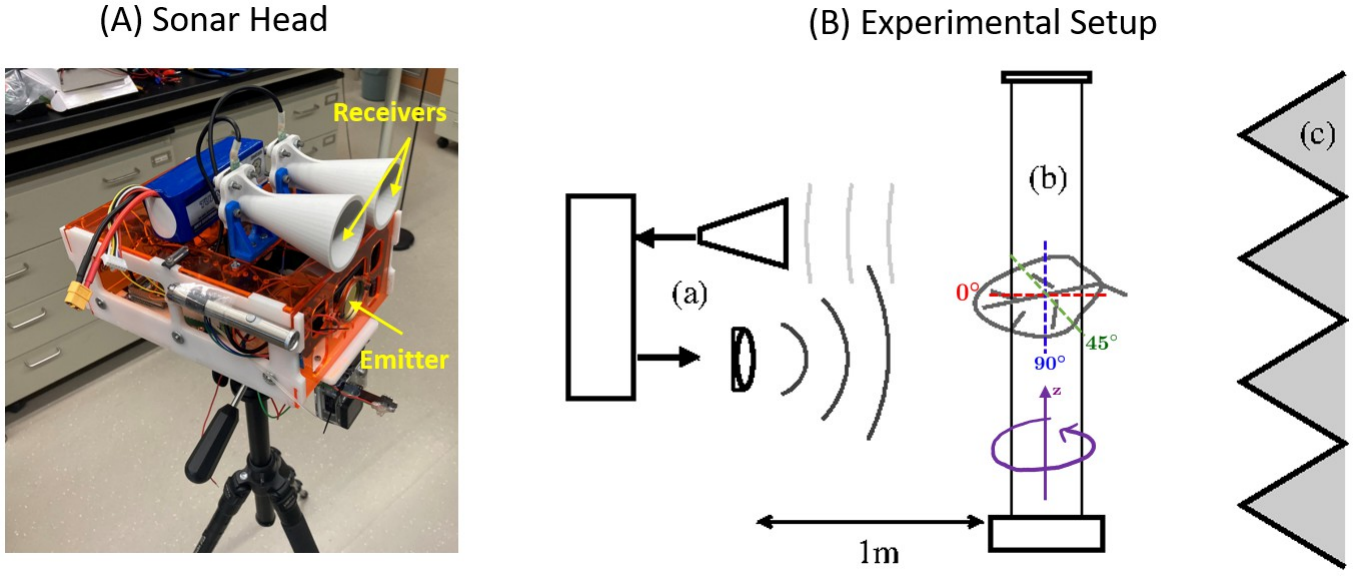
Experimental setup. (A) The sonar head. (B) A diagram of the experiment setup, where (a) denotes a side view of the sonar head, (b) denotes a leaf suspended on thin filament, and (c) denotes the wall of the anechoic chamber. The dashed lines show directions of vertical rotations at 0° (red), 45° (green), and 90° (blue). The purple arrows show the direction of horizontal rotations.

To study validity of the improved foliage echo simulator, we designed an experiment to collect real foliage echoes from single leaf samples of four species of trees. The experiment was performed using a biomimetic sonar head in an anechoic chamber, as shown in Fig 1(A). The sonar head was equipped with an emitter and two receivers, whose locations were marked in Fig 1(A). The emitter was an electrostatic ultrasonic loudspeaker (Series 600 open-face ultrasonic transducer, diameter 38 mm, SensComp, Livonia, MI, USA) with a two-sided -3 dB beamwidth of 10° at 50 kHz. The receivers were two MEMS capacitive microphones (Monomic, Dodotronic, Rome, Italy) with cones attached. Each cone has an outer diameter of 50 mm and a length of 10 cm. The spacing between the two MEMS is 7 cm. The sonar head used a PXIe-6356 data acquisition system (National Instruments, Austin, Texas, USA) with a 500 kHz sampling rate and 16-bits resolution to perform digital-to-analog and analog-to-digital conversion to create the pulse waveform and record the echoes respectively. The emitted pulse consisted of a Hamming window as the envelope and a 2 ms frequency modulation from 20 to 105 kHz. A tripod was used to mount the sonar head at a fixed height.

A diagram of the experiment is shown in Fig 1(B). During the experiment, a single leaf was suspended between two thin filaments (fishing lines) and was positioned one meter away from the sonar head. The fishing lines were connected to a stepper motor which can rotate the leaf horizontally (along the azimuth direction from 0 to 360 degrees about the z axis; direction is shown using purple arrows on Fig 1(B)). When suspending the leaf on the fishing lines, we set three angles (rotated about the leaf normal axis): horizontal, 45 degree tilted or vertical. This gives three vertical angles of the leaf—0, 45 and 90 degrees; directions are shown as red, green and blue dashed lines on Fig 1(B). Under each leaf orientation setup, we record the leaf angles, total length of the leaf, tree species along with the echo signals received from the two microphones of the sonar head.

In addition to the above experiment, we have also carried out experiments to measure beampatterns of the microphone-plus-cone structure of the sonar head. These measurements were then used to construct emitter and receiver beampattern in our refined simulator. When measuring beampattern for the microphone-plus-cone structure, the whole microphone-plus-cone was mounted on a pan-tilt unit (model PTU-E46-17, FLIR Systems, Inc., Burlington, ON, Canada) and rotated over ±25° both in azimuth and elevation with 1°resolution. A loudspeaker was mounted at the same height as the microphone-plus-cone and set one meter away from its center to the microphone. The transmitted signal was a two millisecond constant frequency waveform from 20 kHz to 100 kHz with a step of 5 kHz. A total of 20 repeated measurements at each position for each frequency were made.

To measure the beampattern of the loudspeaker, we mounted the loudspeaker to the pan-tilt and scanned from −45 degrees to + 45 degrees with one degree per step horizontally. A 1/8-inch pressure-field microphone (Brüel & Kjaer, Virum, Hovedstaden) was mounted one meter away from the loudspeaker at the same height. The frequency was set between 20 kHz and 105 kHz with 5 kHz per step. At each position and each frequency, the test was repeated by 10 times. A comparison between beamwidths of the loudspeaker at -3 dB and -5 dB with that from the mathematical piston model gives a good match, so we decided to use the piston model to characterize the beamwidth of the loudspeaker.

The speaker and microphone directivity patterns, both analytical and measured, were approximated in simulation using product of two Gaussian functions, one function each for azimuth and elevation. The standard deviation of both Gaussian functions were set to half of the -3dB beamwidth either intended (as a user input) or estimated based on the beampattern data collected in experiment.

### Data preprocessing, simulation, and comparison

Raw echoes were recorded for single leaf samples from four different tree species: oak, dogwood, yew, and tulip tree. Each recording contained two echo signals, one from each microphone. The raw recordings were 25 milliseconds long with a sampling rate of 400 kHz. The size of the leaves varies from 2.8 to 9.0 inches, the azimuth angle of the leaves varies from 1.8 to 358.2 degrees, and the elevation angles can be either 0, 45, or 90 degrees.

Based on the raw echo recordings, several pre-processing steps were carried out to obtain impulse responses that are comparable with the simulation outputs. First, we bandpass-filtered the emitted chirp with a frequency band ranging from 25 to 95 kHz, a range that matches with the simulation. We then calculated the cross correlation between each echo recording and the filtered chirp, which resulted in an estimate of the impulse response signal. Next, we applied a cut-off and only retained the signals between 5 and 10 milliseconds, a range that covers the impulse responses in all cases. After these steps, we extracted two features to quantify the noise level and used them to filter out noisy measurements. The first feature consists of peaks extracted from the envelope of each impulse response. To obtain this feature, we first obtained the envelope of the impulse response and then detected peaks of the envelopes that are above a pre-specified threshold 0.3. The second feature is the integration of the envelope signal across the time domain. We filtered out noisy recordings based on three conditions: (i) the total number of envelope peaks is greater than three, (ii) the largest time difference between two neighboring peaks are greater than 0.2 miliseonds, and (iii) the area under the normalized envelope signal is greater than 0.2. These pre-processing steps resulted in a total of 8, 695 impulse response measurements, among which 5, 269 are from oak, 2, 416 from dogwood, 180 from yew, and 830 from tulip tree.

We further applied our refined foliage echo simulator to generate single leaf impulse responses in order to compare with experimental data. The simulation parameters were chosen to match the leaf parameters and the experimental setup. Specifically, the distances between sonar and leaf center as well as leaf center and microphone were both set at one meter. The leaf radii and sound incident angles used to generate simulated echoes were also set the same as the experiment. When simulating the frequency domain signal, we set a frequency range from 25 to 95 kHz. In contrast to the previous simulator [15, 16] which assumes a flat frequency response for microphone and speaker, in our simulation, we adopt the real frequency response curves of the microphone and speaker used in the experiments to calculate the emission and reception gains at each frequency. While the above settings were made to best match the experiment, unlike the experiment, we still assumed that there is only one receiver in the simulator and the emitter and receiver were in the same position. Thus, only one impulse response is simulated for each leaf setup.

**Fig 2:**
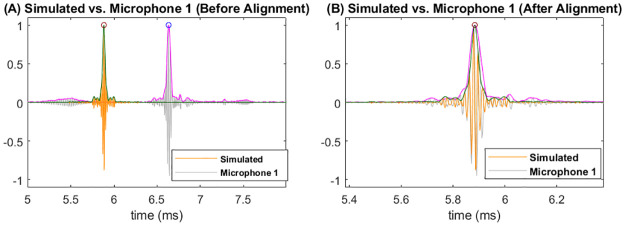
Align simulated and measured impulse responses by matching the highest peak of the envelopes. (A) Before alignment. (B) After alignment.

Before comparing the experimental measurements with simulated signals, several further adjustments were performed. First, the amplitudes of impulse responses for the two microphones are on different scales, which is likely caused by differences in microphone gains. On the other hand, amplitudes of the simulated impulse response are also influenced by the emission/reception setups (e.g., gains, input frequency, etc.) for the emitter and receiver. We thus rescaled each impulse response signal, either simulated or measured through experiment, by dividing its magnitudes by the highest peak of the signal’s envelope. Furthermore, the times of arrival for impulse responses in experiment vary between 5 and 7 milliseconds, which do not align with that of the simulated signal. This misalignment is due to the fact that in experiments, microphones were placed on the two sides of the speaker. Therefore, the distances between microphones and the leaf is greater than one meter, whereas in simulation we have assumed that the speaker and receivers are both one meter away from the leaf. We thus aligned the experimental signals with the simulated ones by matching locations of the highest peak of their envelopes. Specifically, we shift the experimental signal along the time axis so that the location of the highest peak matches that of the simulated signal. We found that rescaling and shifting by using the highest peak of the envelopes gives better alignment than simply using the maximum amplitude of the signal. In Fig 2, we show a simulated signal and an experimental signal, together with their envelopes and the highest peaks, before and after the alignment. The signals shown in Fig 2 were obtained by using leaf size of 5.0 inches, azimuth angle of 318.6 degree and elevation angle of 90 degree. After aligning the signals, we further cut the signals shorter by retaining 200 sampling points before and 199 sampling points after the peak location, resulting in a signal length of 400 (with a time span of 1 milisecond) for all impulse responses. When visualizing the waveforms and calculating descriptive statistics, we used the time argument of the simulated impulse response as the common time argument for both simulated and experimental data.

### Estimation of the surroundings

Comparing simulated echoes with experimental data helps us evaluate the effectiveness of the foliage echo simulator in representing the biosonar ensonification procedure in reality. To further demonstrate the potential application of the foliage echo simulator, we adopted an analytical approach to estimate the surroundings based on the simulated foliage echoes. Such an approach provides evidence that the simulated echoes indeed carry information about the environment, thus can be used for more sophisticated biosonar sensing tasks. It is noteworthy that, though methods for estimating leaf parameters based on simulated echoes have been proposed in recent publications [15, 20], estimation of the surroundings was not considered. In this study, we focus on estimating a rough map of the surroundings and predicting some important features of the foliage.

**Fig 3:**
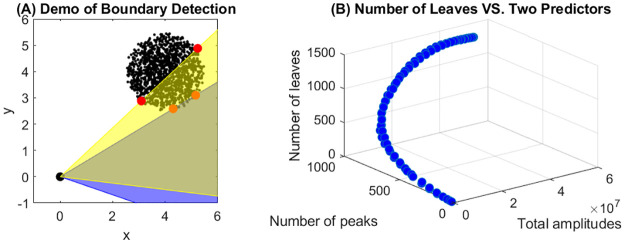
Estimation of the surroundings. (A) A demonstration of detecting boundary points of a ball-shaped leaf cluster at two azimuth angles. (B) Relationship between the number of leaves and two of the predictor variables: the total amplitudes and the number of peaks.

We consider a forest environment consisting of tree foliage, and aim to reconstruct a map of the foliage environment. For this purpose, we calculate distances from sonar to both the nearest and the farthest leaves within the sonar beam by using the arrival times of the echo responses. These distances are then used to determine the boundaries of the foliage. Let *t*_0_ and *t*_1_ denote the onset and end times of the reflections, which can be obtained by identifying the earliest and latest peaks from the echo responses. The distances from sonar to the onset and end reflectors are calculated by *d*_0_ = *ct*_0_/2 and *d*_1_ = *ct*_1_/2, respectively, where *c* is the speed of sound. Note that a “snapshot” of these distances at a specific sonar direction is not sufficient to determine the boundaries of the foliage within the sonar beam. However, by changing the sonar directions continuously along azimuth-elevation, we can collect information about the dynamic change of these distances when the foliage enters and exits the sonar beam. Such dynamic information forms the basis of our foliage boundary identification algorithm. Moreover, in order to resolve difficult non-identifiable situations occasionally (e.g., small gaps between two clusters of tree foliage), it is necessary to vary the beamwidth of the sonar. In Fig 3(A), we demonstrate a 2D example of how the boundary of a ball-shaped leaf cluster can be detected by scanning along the azimuth direction counterclockwise. In this example, both sonar and microphone are located at (0, 0, 0), shown as a black circle. Tree leaves, shown in black dots, are distributed uniformly in a sphere with radius 1.5 and center (4, 4, 0), with 100 leaves per cubic meter. The scan is performed at a angular spacing of 1°. The blue and yellow areas in Fig 3(A) highlight the sonar beams at two different azimuth angles. The red dots mark the onset and end of the leaf reflectors detected based on the minimum and maximum arriving times of echo responses. The edge of the foliage can be detected by connecting the boundary points along the scanning steps. We note that simulation of foliage echoes from the ball-shaped leaf cluster follows the same principle described previously—we need to specify relative locations of each leaf and the sonar, incident angles, and the leaf sizes as inputs. The simulation involves calculating the frequency domain response for each leaf within the sonar beam and summing over across all leaves.

Besides the arrival times, biosonar echoes also carry other information of the reflectors which may be used to estimate important features of the foliage. Previous works [15, 20] have shown that the estimates are often non-unique, i.e., some parameters such as reflector size and orientation appear to be non-identifiable—larger and smaller leaves may result in the same impulse response under different orientations. Therefore, instead of trying to estimate all reflector features using the 1-dimensional impulse responses, we believe it is more reasonable to focus on estimating features that are identifiable and relevant to the task at hand. In this study, we focus on predicting the number of leaves within the sonar beam from the impulse responses. The prediction result can be used to determine the leaf density of the foliage cluster, a key parameter for tasks like path planning—changes in leaf density at different scanning angles may help localize gaps or holes to fly through. Besides the number of leaves, there are a few confounding parameters, such as the mean leaf size and the mean leaf orientation. These confounding parameters are relevant to factors such as tree species, and may be determined by implementing classification algorithms such as [23]. In this study, we treat the confounding parameters as fixed during the training and prediction procedure. We selected three types of features as predictors, including the sum of envelope’s amplitudes (which we call “total amplitudes”), the number of peaks of the impulse response, and the quantiles of heights of the peaks. Fig 3(B) demonstrates the relationship between the number of leaves and two of the predictor variables, the sum amplitudes and the number of peaks, based on data obtained in the scanning procedure shown in Fig 3(A). From Fig 3(B), we observe a positive monotonic and non-linear relationship between the number of leaves and the two predictor variables.

## Results

We compared experimental impulse responses with outputs from the foliage echo simulator using several approaches. First, we visualized the waveforms by plotting the aligned signals on a common time domain. We then measured the similarity by calculating the normalized cross-correlation—the correlation coefficient between one discrete signal and the shifted (lagged) copies of another signal, recorded as a function of the lag. If two signals are identical, then the normalized cross-correlation equals one at lag zero, otherwise it lies between zero and one. We use the maximum value of the normalized cross-correlation as a statistic to summarize the amount of similarity. In addition to the time domain comparison, we also compared the signals in frequency domain by plotting the single-sided amplitude spectra. We use the L2 distance (integral of squared difference) between the amplitude spectra as a statistic to summarize the similarity in frequency domain.

We performed three groups of pairwise comparisons between impulse responses obtained from the two microphones and the simulation—microphone 1 vs. 2, microphone 1 vs. simulated, and microphone 2 vs. simulated. The similarity between signals from microphones 1 and 2 serves as a benchmark, which represents an ideal situation under which no systematic differences between the two signals exist. We expect the differences between experimental measurements and simulation outputs to be larger than the benchmark due to simplifications and approximations made in the foliage echo simulation model.

**Fig 4:**
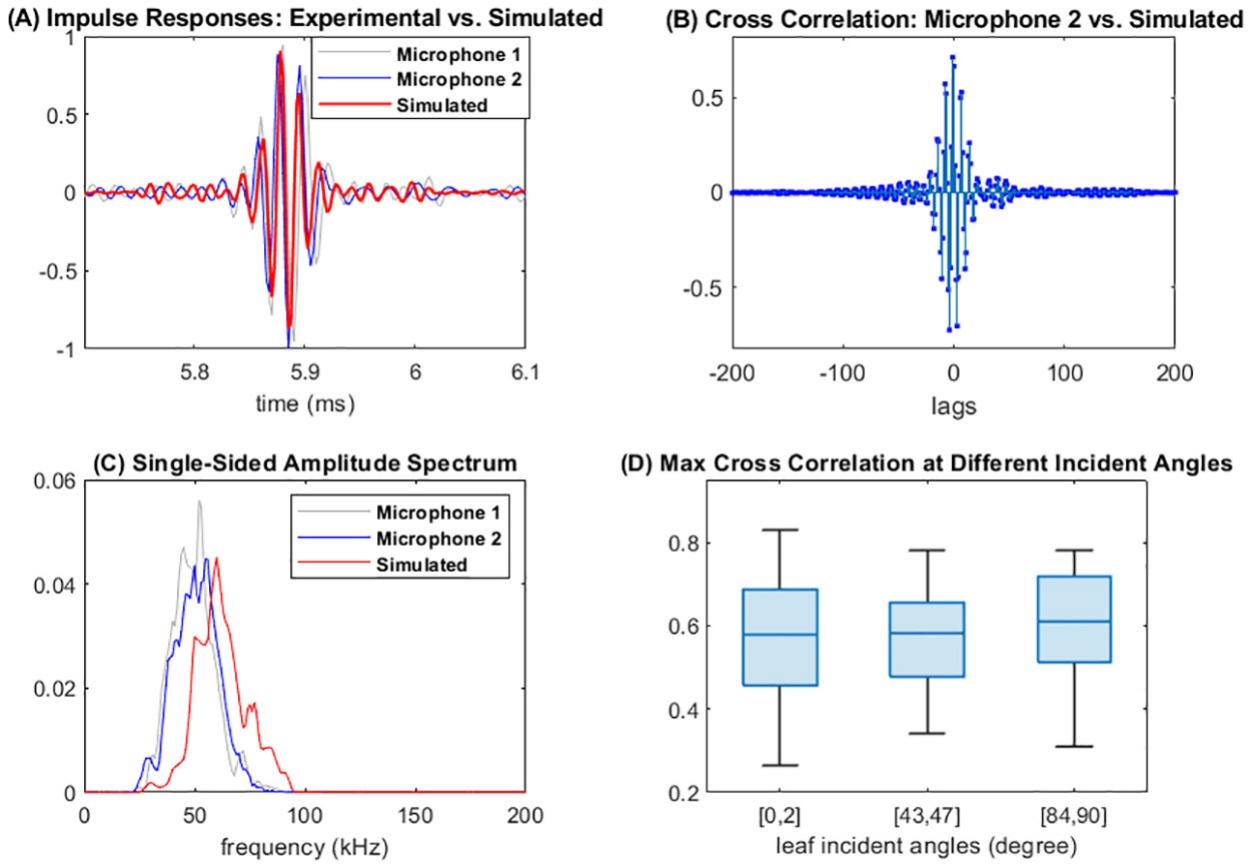
Comparison plots for one measurement. (A) Waveforms of impulse responses from the two microphones and simulation. (B) The normalized cross correlations between microphone 2 and simulated signal, plotted as a function of lags. Here, the lag refers to the number of phase shift positions. (C) Frequency domain comparison—single sided amplitude spectra for impulse responses from the two microphones and simulation. (D) Boxplots of the maximum absolute cross correlation at three different incident angle intervals.

In Fig 4(A)–4(C), we demonstrate comparison plots for one measurement about a leaf from the oak tree. From Fig 4(A), we see that waveforms of the impulse responses from the two microphones and simulation are similar. Such similarity is also observed for other measurements with slight variation. Fig 4(B) plots the normalized cross correlation between impulse responses from microphone 2 and simulation. The benchmark cross correlation between two microphones shows a similar pattern but with a higher peak value. Fig 4(B) shows that after alignment, the peak of normalized cross correlation appears at lag −4, with a (absolute) peak value 0.7225 which is close to the benchmark value between the two microphones (0.8621). Fig 4(C) shows the frequency domain comparison—the amplitude spectra of signals from the two microphones and the simulation. We observe that while the overall shapes are similar, the amplitude spectrum for simulation appears shifted to the right, with lower signal power in the frequency range of [20, 50] kHz and higher signal power in the range from 65 to 95 kHz. For this example, the pairwise L2 distances for the amplitude spectra are 0.0025 (microphones 1 vs. 2), 0.0223 (microphone 1 vs. simulation), and 0.0135 (microphone 2 vs. simulation). To further demonstrate how the comparison results change when the leaf angles is changed, in Fig 4(D) we demonstrate boxplots of the maximum absolute cross correlation at three different incident angle intervals [0, 2], [43, 47], and [85, 90] degrees. From Fig 4(D) we do not observe evident differences across the three intervals. To verify this observation statistically, We also performed a one-way analysis of variance (ANOVA) to test whether there are systematic differences on means of the three groups, which gives a p-value of 0.1779. Therefore, we conclude that there are no significant differences on the means of the three groups.

To quantitatively summarize results of comparison, we extracted two statistics from each pair of comparison, the maximum absolute value of the normalized cross correlation in time domain and the L2 distances of the single sided amplitude spectra in frequency domain. The mean values as well as the 2.5% and 97.5% percentiles calculated across all measurements for each species are listed in Table 1. From Table 1, we observe that the cross correlation between experimental data and simulated one are lower than the benchmark values between the two microphones, and the statistics for microphone 1 vs. simulation and microphone 2 vs. simulation are similar. Similar pattern is also observed for the L2 distances in frequency domain, i.e., distances for microphone 1 vs. simulation and microphone 2 vs. simulation are close, and both are higher than the benchmark values. These patterns are consistent with our expectation—the simulator will generate data with larger differences than the benchmark due to additional simplifications (e.g., on leaf shape, beam patterns) made. Looking across the tree species, we found that most statistics are similar for all tree species, except that for the yew tree, the statistics between experimental and simulated signals give slightly lower cross correlations and higher L2 distances than the other three species. This may be related to the fact that the long, narrow shape of the yew leaf deviates further from the disc-shape assumption made in the simulation, causing larger approximation error in simulation. Overall, the statistics in Table 1 implies that while showing some deviations from the benchmark, differences between the experimental and simulated data are still on the same scale with the benchmark thus are not too far off the ideal situation.

**Table 1:** Summary statistics for comparison.

		Mic 1 vs. Mic 2	Mic 1 vs. Sim.	Mic 2 vs. Sim.
Cross Corr.	Oak	0.786 (0.506, 0.933)	0.564 (0.323, 0.772)	0.560 (0.288, 0.791)
	Dogwood	0.778 (0.457, 0.923)	0.584 (0.364, 0.799)	0.606 (0.370, 0.783)
	Yew	0.718 (0.403, 0.893)	0.564 (0.388, 0.802)	0.405 (0.209, 0.583)
	Tulip Tree	0.797 (0.526, 0.947)	0.582 (0.318, 0.785)	0.579 (0.315, 0.788)
L2 Dist.	Oak	0.009 (0.001, 0.031)	0.024 (0.009, 0.047)	0.028 (0.010, 0.058)
	Dogwood	0.010 (0.002, 0.034)	0.024 (0.008, 0.046)	0.025 (0.011, 0.046)
	Yew	0.019 (0.006, 0.051)	0.025 (0.007, 0.041)	0.049 (0.024, 0.079)
	Tulip Tree	0.007 (0.001, 0.026)	0.022 (0.009, 0.049)	0.024 (0.009, 0.054)

Cross Corr: the maximum absolute value of normalized cross correlation; L2 Dist: the L2 distances of single sided amplitude spectra; Mic 1: signal from microphone 1; Mic 2: signal from microphone 1; Sim: the simulated signal.

**Fig 5:**
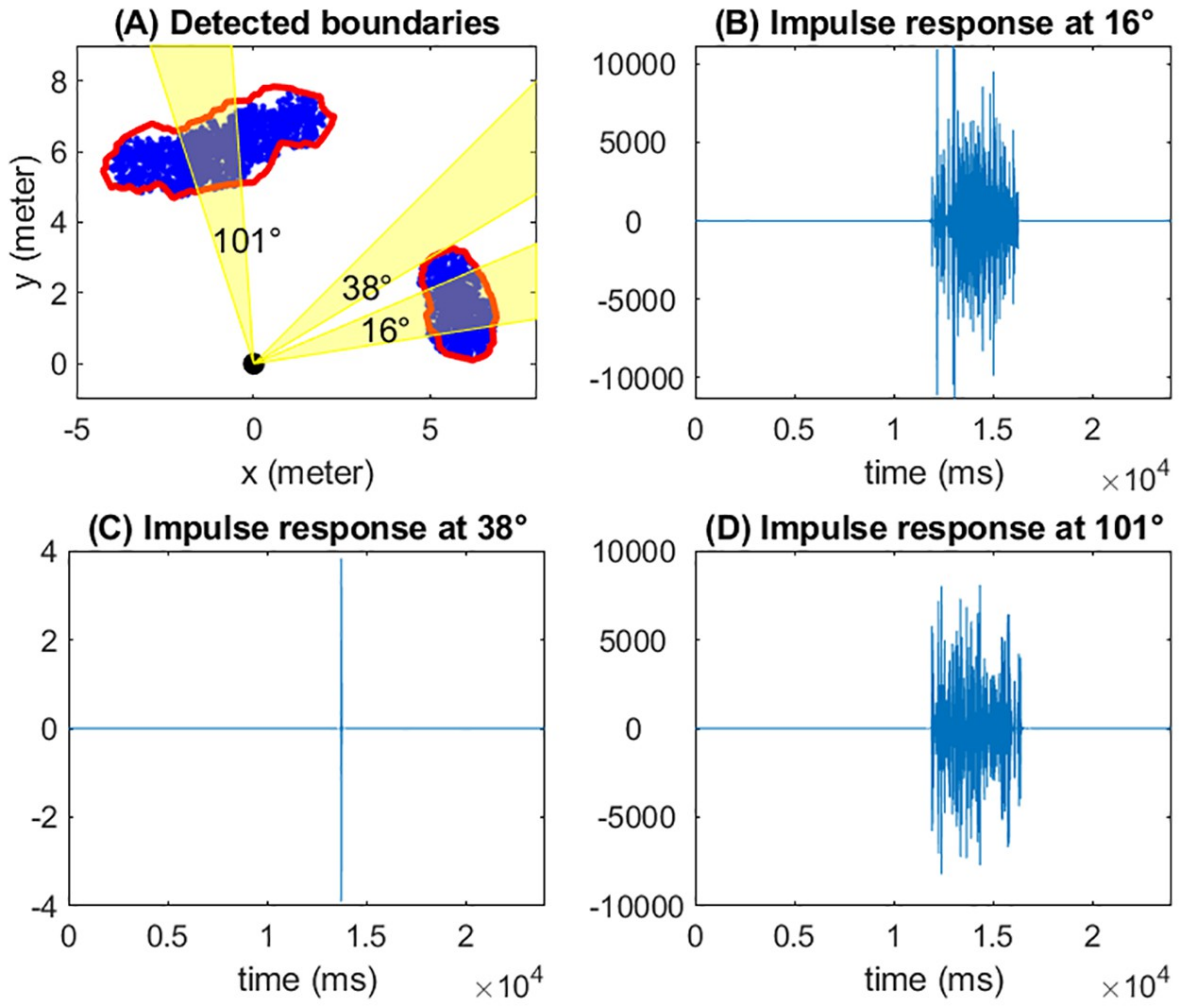
Boundary detection. (A) An overlooking view of two isolated clusters of foliage, together with their detected boundaries. For both emission and reception, the beampattern peak amplitude is set to 40 dB, and the beamwidth is set to 5°. Tree leaves are shown in blue dots, and the detected boundaries are shown in red lines. Sonar beams at three azimuth angles 16°, 38°, and 101°are shown in yellow color. (B,C,D) The corresponding echo waveforms at 16°, 38°, and 101°.

To illustrate how the simulated foliage echoes can be used to estimate the surroundings, we simulated an environment that consists of two isolated clusters of foliage. Each cluster contains multiple spherical regions filled with leaves, with radii ranging from 0.8 to 1 meter. Within each spherical region, leaves are uniformly distributed with density 100 per cubic meter. However, throughout the entire cluster leaves are not uniformly distributed because of the overlap of the spherical regions. The leaf sizes are approximated by disks whose radii follow a truncated Gaussian distribution with mean 0.05 meter and standard deviation 0.005. A top view of such an environment is shown in a 2D plot in Fig 5. The sonar was put at the origin [0, 0, 0], about 6 to 7 meters away from the centers of these spherical regions. For simplicity, we fixed the elevation angle at 0° and use the sonar to scan the environment along the azimuth direction on range [−30°, 180°] with step size 1°. The beamwidth of the sonar was set to 5° which enables us to identify the gap between the two clusters. The estimated foliage boundary is marked by red lines in Fig 5. From this figure, we see that the foliage edge can be detected with reasonable accuracy by using the arrival times.

**Fig 6:**
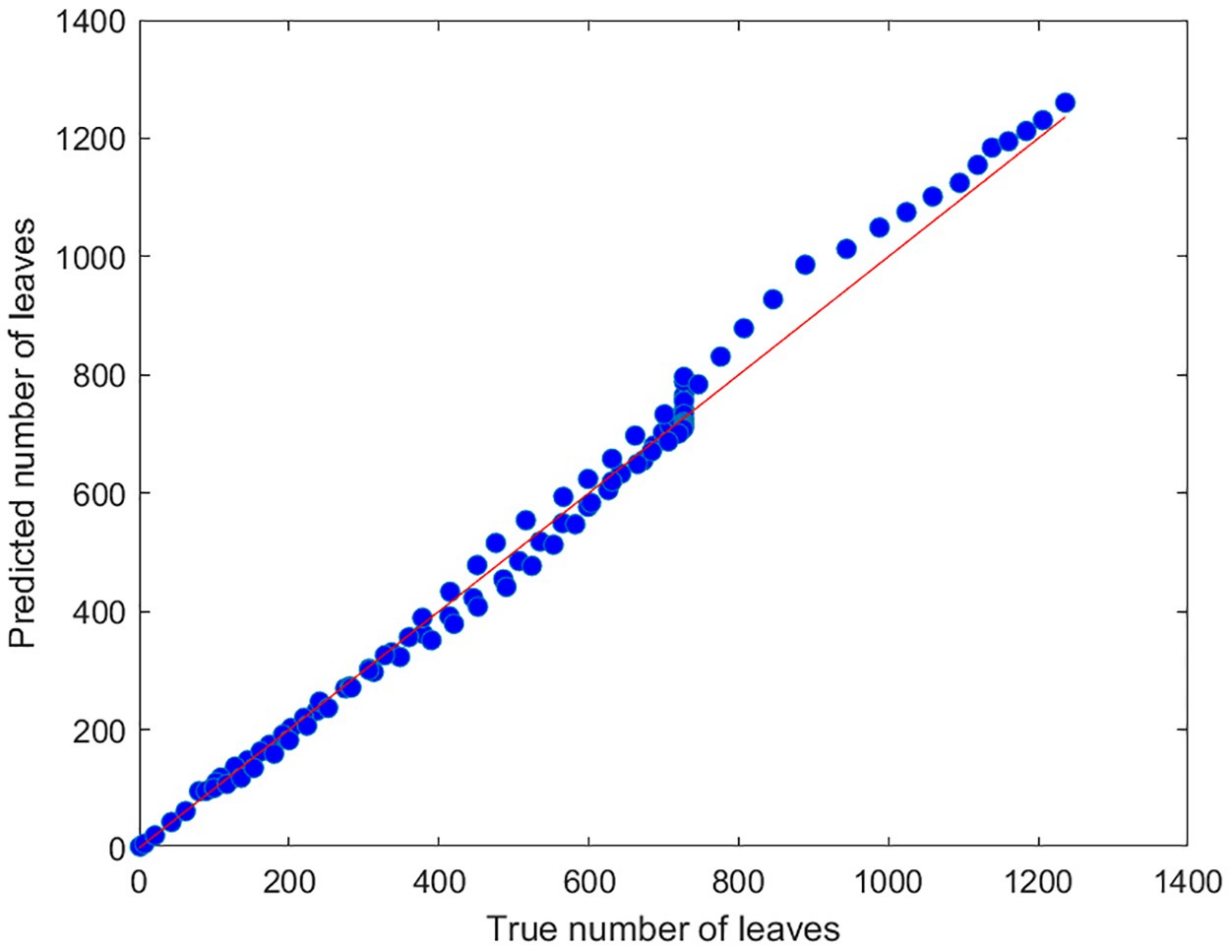
Prediction of number of leaves in test data. The number of leaves is predicted by a function fitting neural network with one hidden layer of 20 neurons. Inputs of the neural network include 13 predictors from the impulse response, including the total amplitudes, the number of peaks, as well as the (0, 10, …, 100)% quantiles of the peak heights. The red line represents *y* = *x*.

In addition to estimating the boundary, we trained a model to predict the number of leaves in the sonar beam during the scanning process using the same simulation setup as in boundary detection. A total of 13 predictors from the impulse response were used, including the total amplitudes, the number of peaks, as well as the (0, 10, …, 100)% quantiles of the peak heights. We adopted an artificial neural network model for training and prediction. This model incorporates a function fitting neural network with one hidden layer of 20 neurons. Training and test data are generated under the simulation scene that consists of two isolated clusters of foliage. The training data are obtained from scanning of 100 simulated scenes with leaf sizes and orientations randomized but spherical centers and radii fixed. The test data consist of one scan of a simulated scene with both leaf parameters and spherical centers and radii randomized. During the training stage, data on the 13 predictors and the number of leaves at each azimuth angle in [−30°, 180°] were collected during each step of the scanning process and fed into the neural network. The trained neural network was then used to predict the number of leaves in the test data. For comparison purpose, data from one of the total 100 scans in the training data was chosen as validation data. We found that, while the mean number of leaves in the training data is 630.99, the mean absolute error (MAE) of the prediction for the validation data is as low as 15.59. In contrast, the prediction MAE for the test data is 20.85. The true and predicted number of leaves in the test data are compared in a scatter plot in Fig 6. These results demonstrate that the simulated foliage echos carry sufficient information for estimating the number of leaves in the sonar beam during the scanning process.

## Discussion

We have carried out a validation study to demonstrate the effectiveness of the foliage echo simulator of [15, 16] in reflecting real echo-generating process. We have refined the existing simulator in order to introduce more flexibility and allow a better match with the experimental setup. We used multiple summary statistics to characterize the differences between simulated and experimental impulse responses on single leaves. We treat the differences between signals received from two microphones in experiments as the benchmark results, and found that simulated impulse responses does not deviate from the experimental measurements substantially. This indicates that the computational foliage models are able to capture most salient features of the realistic foliage echo. In addition to comparing with experimental data, we also proposed a statistical approach to estimate the surroundings based on simulated echoes. This approach can be generally applied to more complicated sensing scenarios such as UAV path planning.

While not all features of the refined foliage echo simulator have been used in the setup of current study, the additional features make this simulator more general and could contribute to other novel applications in future studies. For example, the refined simulator allows us to place sonar speaker and microphone at arbitrary locations, this enables the study of Doppler shifts during the movement of sonar as well as the Doppler shift compensation mechanism of bats. Furthermore, with more flexibility on sonar directivity, the refined simulator makes it easier to study beam-direction control during situations like obstacle avoidance or contour following.

Our experiment has focused on single leaves, which allows us to match the simulation with the experiment as close as possible. Since the impulse response of multiple leaves is simply the superposition of those from single leaves, as long as the simulator is valid for single leaves, it is probably also valid for multiple leaves. Of course this ignores the effect of shading which is not considered in the current foliage echo model. In the former study of [16], experimental data have been collected to study the acoustic effect of shading; they found that shading between two leaves could attenuate the magnitude of impulse responses and this attenuation depends on the relative distances between the two leaves. An interesting future work would be to model the shading effect by introducing an adjusted attenuation function in the foliage echo simulator.

In addition to modeling the shading effect, two other studies can be carried out in the future to further validate effectiveness of the current foliage echo simulator and the estimation approach: (1) A new study to confirm effectiveness of the improved foliage echo simulator on leaf clusters. This requires collecting experimental and simulated data under the similar or closely matching conditions, and comparing them using quantitative methods. (2) A new study to further test performance of the proposed approach on estimating partial maps and predicting the number of leaves by using experimental data. This requires collecting experimental data in a scene that contains multiple leaf clusters, and applying the proposed estimation and prediction approach solely based on experimental data.

Finally, we have primarily focused on the forest environment. In addition to leaves, natural surroundings may have many other reflectors such as branches, rocks, and water. The contribution of these different reflectors may vary under different situations. Considering a forest that involves thousands of leaves in front of branches, modeling only the leaves is probably a fair approximation. However, it is reasonable to expect that such assumption may need to be relaxed for different outdoor situations.

Data used in this paper have been made available online [24].
